# Breast metastasis of pulmonary pleomorphic carcinoma: a case report

**DOI:** 10.1186/s40792-017-0302-6

**Published:** 2017-02-10

**Authors:** Tomoyuki Fujita, Hajime Nishimura, Ryoichi Kondo, Kinya Furukawa, Yukio Morishita, Minoru Fujimori

**Affiliations:** 1 Department of Breast Surgery, Tokyo Medical University Ibaraki Medical Center, 3-20-1 Chuo, Ami, Inashiki, Ibaraki 300-0395 Japan; 2 Department of Breast Surgery, Koyama Memorial Hospital, 5-1-2 Kuriya, Kashima, Ibaraki 314-0030 Japan; 3 Department of Thoracic Surgery, Tokyo Medical University Ibaraki Medical Center, 3-20-1 Chuo, Ami, Inashiki, Ibaraki 300-0395 Japan; 4 Diagnostic Pathology Division, Tokyo Medical University Ibaraki Medical Center, 3-20-1 Chuo, Ami, Inashiki, Ibaraki 300-0395 Japan

**Keywords:** Breast metastasis, Lung cancer, Pulmonary pleomorphic carcinoma

## Abstract

**Background:**

Lung cancer rarely metastasizes to the breast, and breast metastasis of pulmonary pleomorphic carcinoma has not been previously reported.

**Case presentation:**

The patient was a 66-year-old woman who became aware of a mass in the right breast and visited a physician. She was referred to our department for close examination, upon which she was diagnosed with double cancer (right breast cancer and left lung cancer). Needle biopsy findings for the mammary tumor were similar to those for the lung biopsy specimen, but spindle cell or metaplastic carcinoma were possibilities. The initial diagnosis was primary breast cancer. Left upper lobectomy and lymph node dissection were performed for left lung cancer. Both the lung and mammary tumors grew rapidly during the wait for surgery. The white blood cell count was within the normal range at the first examination, but was markedly increased and remained at a high level after surgery for lung cancer. Preoperative chemotherapy was initially planned for the mammary tumor, but surgical treatment was selected in consideration of the clinical course, and right mastectomy and full thickness skin graft were performed. However, the disease rapidly aggravated and the patient died 5 months after the first examination.

**Conclusion:**

The final diagnosis was pulmonary pleomorphic carcinoma with metastasis to the breast on postoperative histopathological examination. We describe this case as the first reported example of breast metastasis of pulmonary pleomorphic carcinoma.

## Background

Lung primary pleomorphic carcinoma is a relatively rare tumor that accounts for about 0.3% of all lung cancer cases and has a poor prognosis [1]. Lung cancer metastasizes to distant regions such as the liver and brain, but rarely metastasizes to the breast. We encountered a patient who was diagnosed with double cancer (breast and lung cancers) before surgery. Left upper lobectomy and right mastectomy were performed, and the lesions were subsequently histopathologically diagnosed as pulmonary pleomorphic carcinoma with metastasis to the breast. We report this unique case with a literature review.

## Case presentation

The patient was a 66-year-old woman with a chief complaint of a mass in the right breast. She had become aware of the mass and visited a physician. A lung tumor was noted on CT, and the patient was referred to our department for suspected double cancer (lung and breast cancers). The patient was diagnosed with double cancer (right breast and left lung cancers) on close examination. Her medical history was unremarkable, but she had smoked 20 cigarettes/day from age 20 to 64 years old. Her first menstruation was at 13 years old and 52 years old at menopause. She had two deliveries. She had no relevant family medical history.

A hard mass of 2 cm in size was palpated in the B region of the right breast. The mass was mobile. The surface was relatively smooth, and the boundary was relatively unclear. The axillary and supraclavicular lymph nodes were not palpable. Blood counts and biochemistry findings were within normal ranges. CEA was high (9.3 ng/mL), but CA15-3, proGRP, and CYFRA were within their normal ranges. Serum G-CSF was as high as 84.0 pg/mL (standard value ≤39.0 pg/mL).

**Fig. 1:**
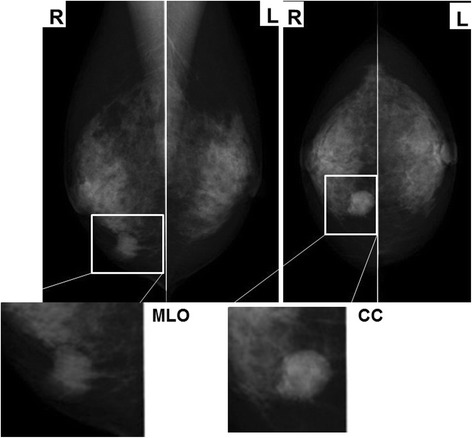
Mammography showing a shadow of an intense irregular mass in the inferior medial region of the right breast

**Fig. 2:**
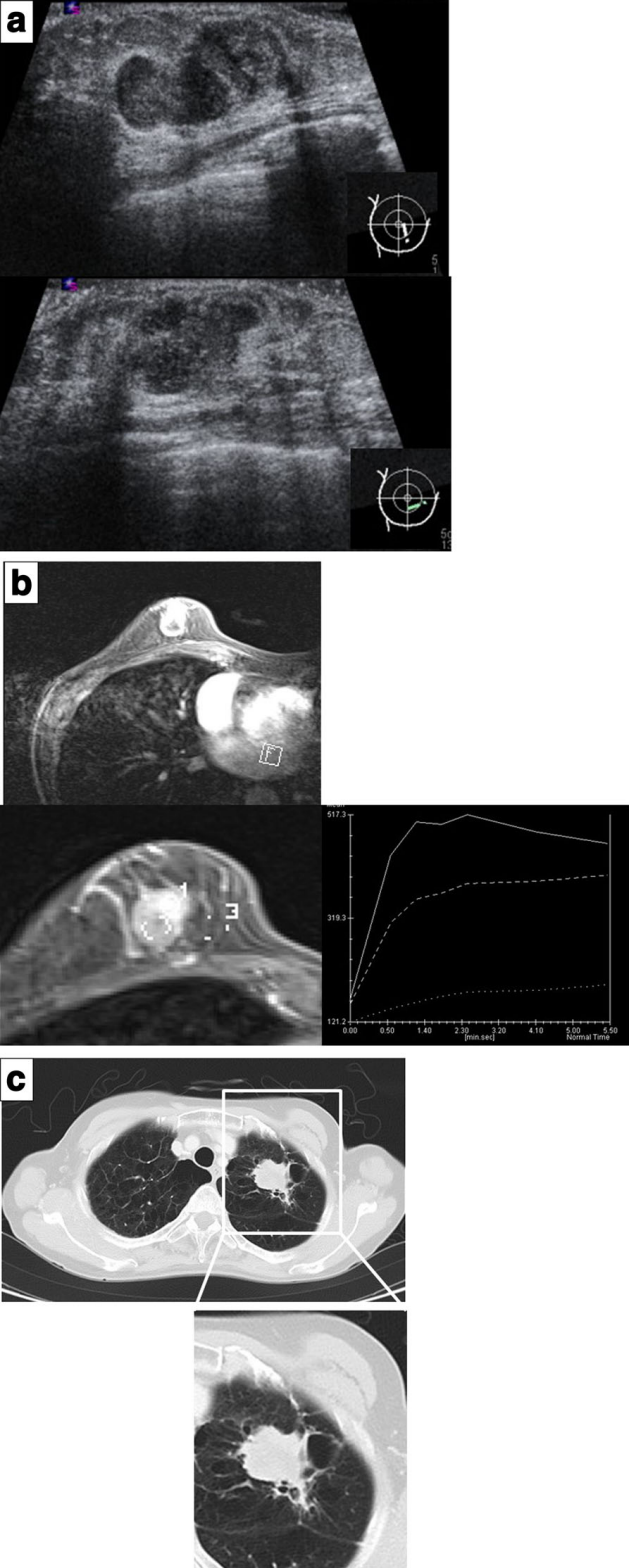
**a** On mammary ultrasonography, an extensive lobular lesion of 12 × 24 × 17 mm with a clear coarse boundary was observed in the right B/D region. **b** On mammary MRI, a contrast-enhanced mass was observed from the early phase. **c** On chest CT, an irregular tumor that was suspected to be lung cancer was present in the left upper lobe

Mammography showed a shadow of an intense irregular mass in the inferior medial region of the right breast and highly suggestive of malignancy as BI-RADS category 4. The left breast was categorized as BI-RADS category 1 (Fig. 1). In mammary ultrasonography, an extensive lesion of 12 × 24 × 17 mm was observed in the right B region. The mass was lobular with a clear coarse boundary. The internal echo was hypoechoic and heterogeneous (partial hemorrhage was suspected), and the posterior echo was enhanced (Fig. 2a). The lesion was hypervascular on color Doppler (data not shown). In MRI of the mammary gland, a mass of 24 mm in size was detected in the right B region. The time-intensity curve was steep, and contrast enhancement was observed from an early phase (Fig. 2b). Chest CT showed an irregular tumor in the left upper lobe, which was suspected to be lung cancer (Fig. 2c). There was no liver metastasis. Bone scintigraphy showed no abnormal accumulation suggesting metastasis.

Histologic findings on needle biopsy specimens of the lung tumor suggested that the lesion was poorly differentiated carcinoma. Assuming that the tumor originated from the lung, it was considered likely to be pleomorphic carcinoma. However, its histology was similar to that in a biopsy specimen collected from the right mammary gland (see below), indicating possible metastasis of the mammary tumor.

Histologic findings on needle biopsy specimens of the mammary tumor suggested that the lesion was assumed to be carcinoma. Spindle cell carcinoma and metaplastic carcinoma were possible, but the histology of the tumor was similar to that observed in the left lung biopsy specimen, suggesting that the lesion had the same origin as that of the lung tumor.

In a preoperative histological examination after diagnosis, the histology of the tumors in the mammary gland and lung were found to be similar, but there was a possibility that the mammary tumor was spindle cell or metaplastic carcinoma, and the lesions were initially diagnosed as double cancer of primary breast cancer and non-small cell lung carcinoma (cT2N1M0 Stage IIA).

**Fig. 3:**
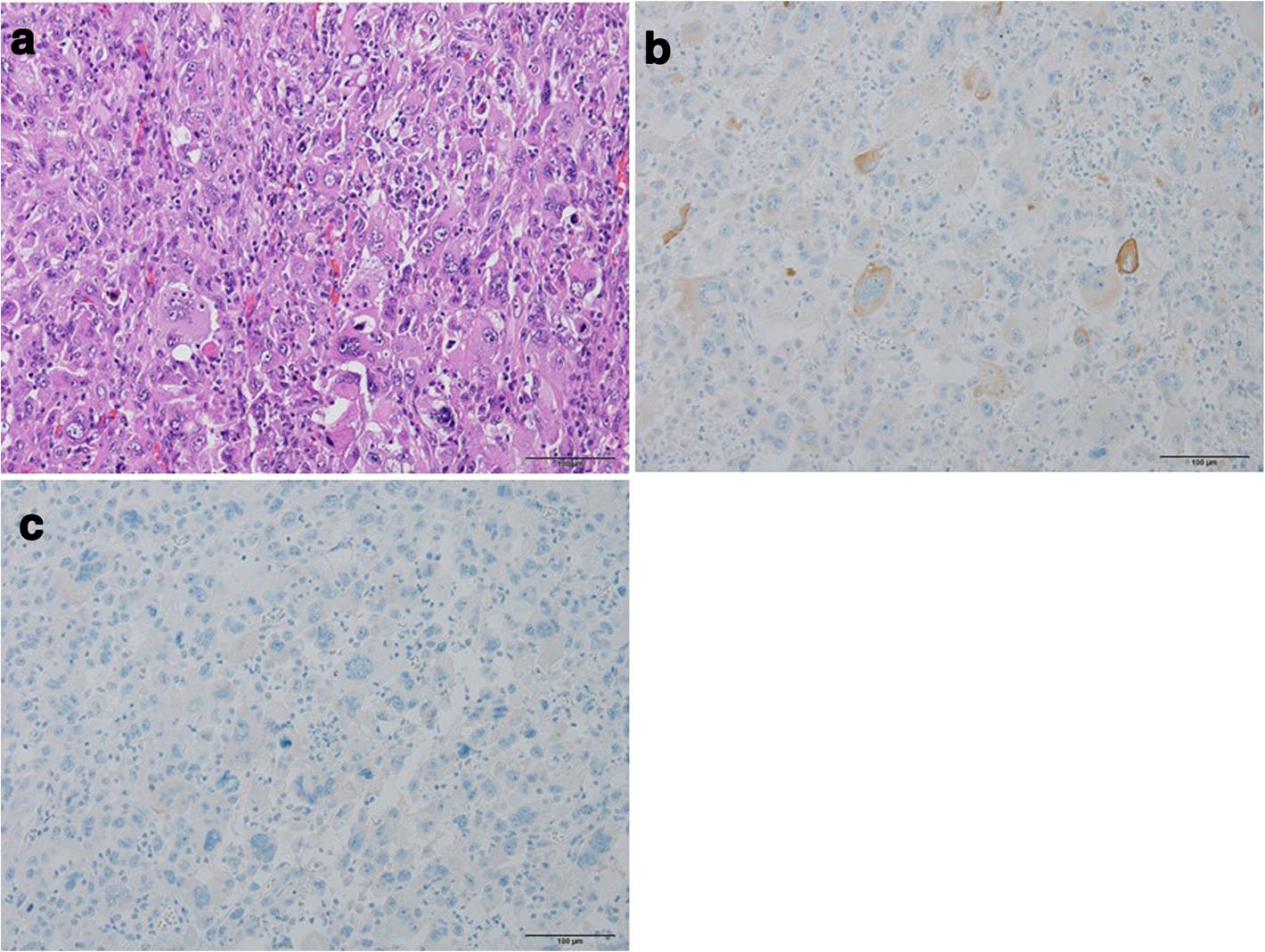
Histology of the lung tumor. **a** Hematoxylin and eosin stain, ×200. Lung tumor of the present case exhibits dyscohesive growth of spindle, oval, or polygonal tumor cells. Multinucleated giant cells are scattered. **b** AE1/AE3 immunohistochemistry, ×200. AE1/AE3-positive tumor cells are scattered. **c** ER immunohistochemistry, ×200. There are no ER-positive tumor cells

Surgery for the lung tumor of left upper lobectomy and lymph node dissection was performed first. Histologically, the lung tumor had dyscohesive proliferation of spindle, oval, or polygonal cells and scattered multinucleated giant cells (Fig. 3a). Immunohistochemically, these tumor cells are partially positive for AE1/AE3 (Fig. 3b), and negative for TTF1 and ER (Fig. 3c). In addition, the tumor had a few foci of squamous cell carcinoma. Furthermore, the patient had intrapulmonary and lymph node metastasis. If the tumor originated from the lung, these findings suggested pleomorphic carcinoma, but the histology was similar to that of the needle biopsy specimen of the right mammary tumor; therefore, the possibility of breast cancer metastasis could not be ruled out. Assuming primary lung cancer, the diagnosis was LU, B1 + 2, 38 × 37 × 60 mm, pl0, D0, G4, Ly0, V1 (EVG), PLC (not tested), pm1, and pN2a-1(6/9).

After surgery for the lung tumor at 1 month after the initial examination, surgery on the mammary gland was performed. The white blood cell count was within the normal range at the time of the first examination, but markedly increased after the lung surgery and remained at a high level. Preoperative chemotherapy was initially planned for the mammary tumor, but surgical treatment was selected based on the clinical course and histopathological diagnosis. After surgery of right mastectomy and full thickness skin graft, the mammary tumor was diagnosed as right breast cancer (cT3N0M0, Stage III). Axillary lymph node dissection was omitted.

**Fig. 4:**
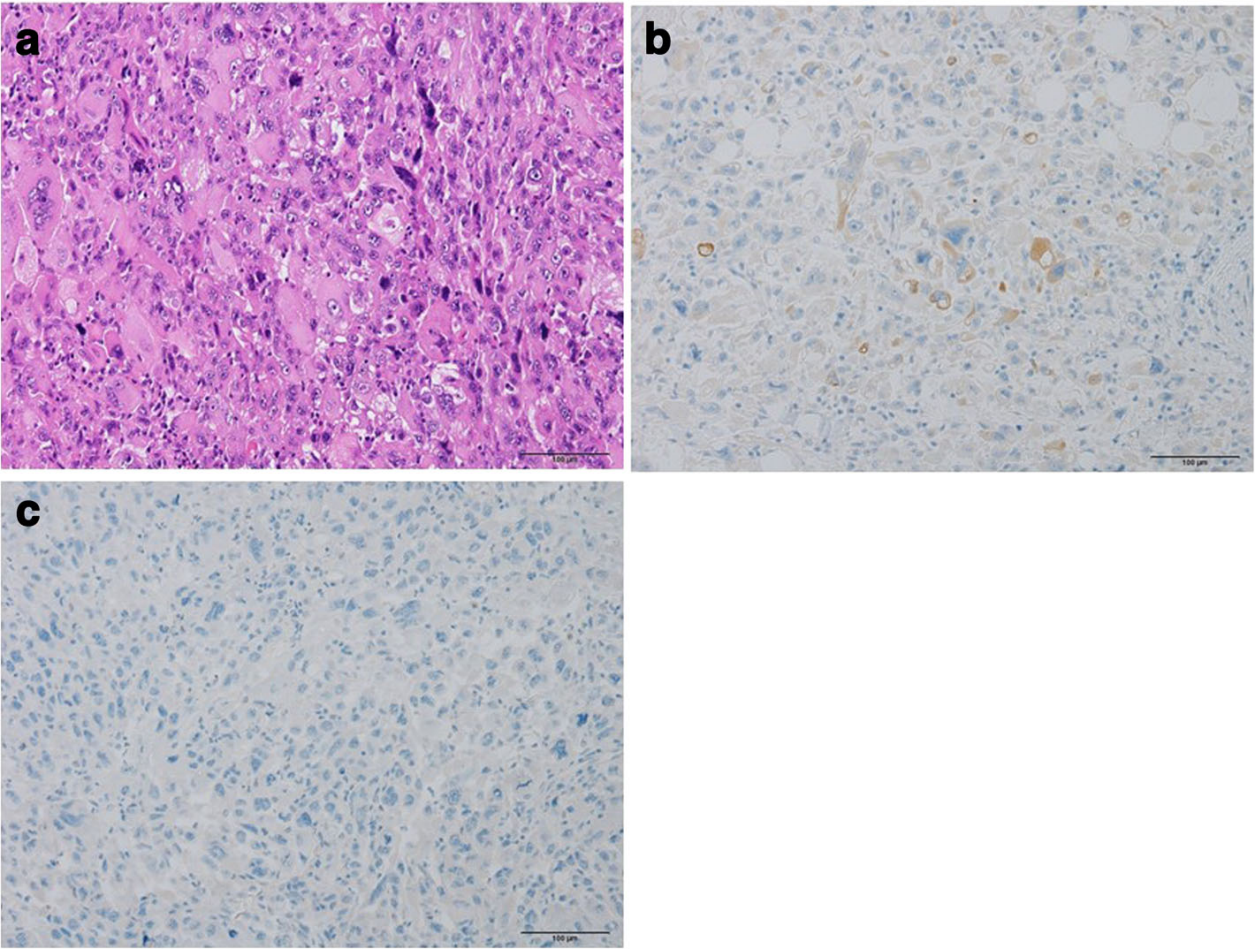
Histology of the breast tumor. **a** Hematoxylin and eosin stain, ×200. The histologic appearances of breast tumor are similar to those of the lung tumor. **b** AE1/AE3 immunohistochemistry, ×200. AE1/AE3-positive tumor cells are scattered. **c** ER immunohistochemistry, ×200. There are no ER-positive tumor cells

The mammary lesion was thought to be a solid tumor that was exposed to the skin. Histopathological findings for the surgically excised mammary specimen are similar to those of the lung tumor (Fig. 4a). However, a histologic appearance of squamous cell carcinoma is not identified. There were no typical features of conventional breast cancer and ductal carcinoma in situ. Immunohistochemically, mammary tumor cells are partially positive for AE1/AE3 (Fig. 4b), and negative for ER (Fig. 4c), PgR, HER2. Ki-67/MIB1 index of tumor cells are ≥90%.

**Fig. 5:**
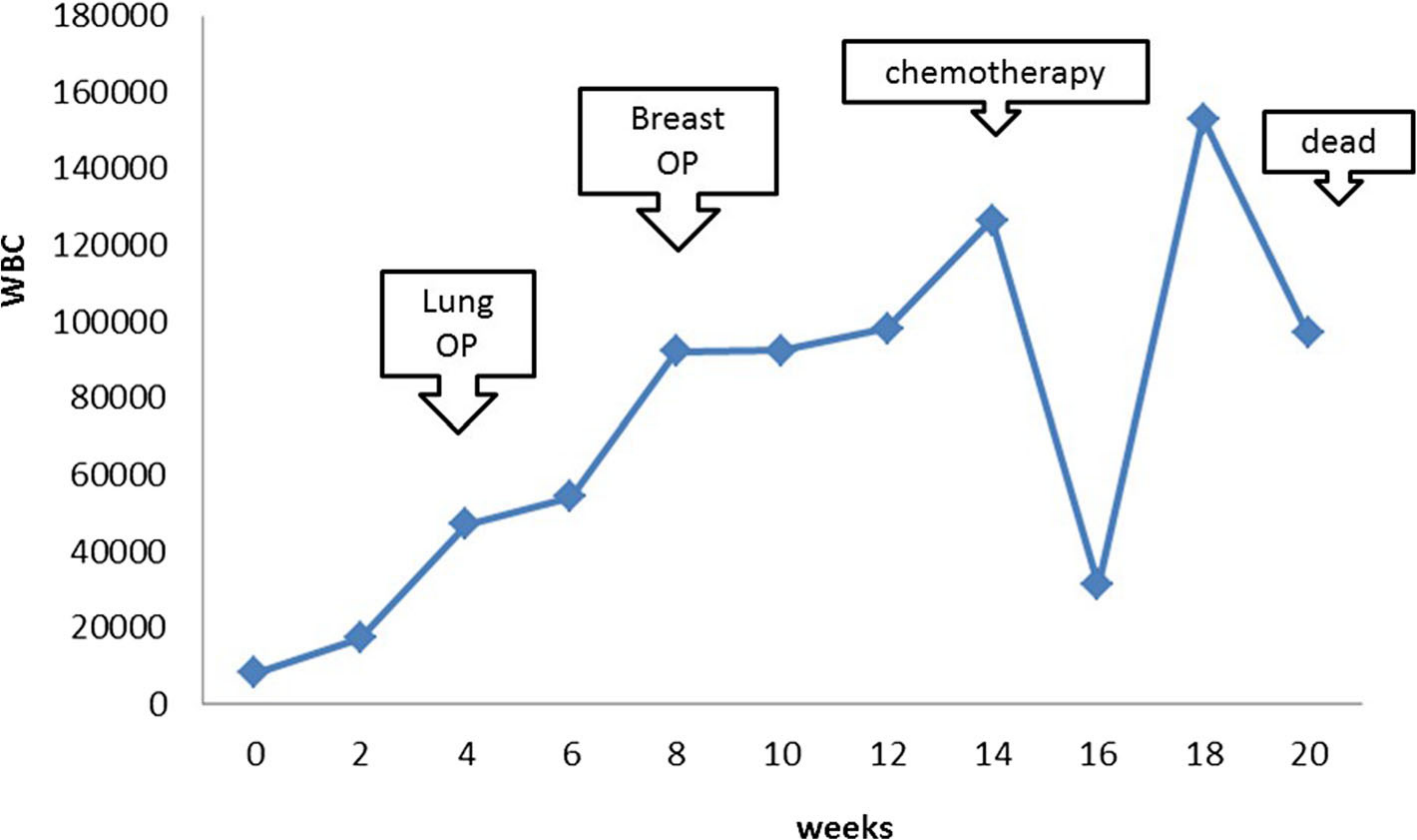
White blood cell counts over the disease course. The counts were abnormally high, corresponding to the disease state

In the course after mastectomy, lower abdominal distension occurred during the hospital stay. A 12-cm giant mass was detected in the pelvis on imaging and diagnosed as a metastatic ovarian tumor. Bilateral pleural effusion, peritoneal dissemination, and findings suggesting bone metastasis were also observed. Chemotherapy with paclitaxel and carboplatin was performed about 1 month after mastectomy, but the ovarian tumor size was not reduced. Systemic conditions aggravated with disease progression, and the treatment was discontinued after the second cycle. The white blood cell count was abnormally high during the course, then transiently decreased after chemotherapy, but subsequently increased again (Fig. 5). Palliative care was performed for disease aggravation, but the patient died about 5 months after the first examination (about 3 months after mastectomy).

## Conclusions

Breast metastasis of cancer from organs other than the mammary gland is rare, and accounts for only 0.5–2% of all cases of malignant mammary gland tumors in Western countries [2]. In Japan, only five cases of breast metastasis of lung cancer have been reported [3–7], including four cases of recurrence after surgery for primary lung cancer [3, 5–7]. In the other case, metastases to the breast and multiple organs were observed in the first examination [4]. There are no characteristic imaging findings for differentiation of primary breast cancer and breast metastasis, and thus histopathological examination is useful for diagnosis.

The general features of breast metastasis of lung cancer include (1) histology similar to that of the primary lesion, (2) infiltrative proliferation of cancer cells around the duct and lobule of the mammary gland, and (3) no cancer in the duct or lobule and retention of the characteristic mammary gland structure [8]. The current case has these three features.

A systematic review of 43 cases of breast metastasis of lung cancer was published in 2014 [8]. These included 33 cases of histologic type non-small cell carcinoma and 10 cases of small cell carcinoma. The series included no case with metastasis of pleomorphic carcinoma. The mean age at the time of diagnosis, ratio of males, and cigarette smoking rate in non-small cell vs. small cell carcinoma cases show no significant differences. Metastasis of non-small cell carcinoma was metachronously discovered after treatment such as surgery in 67% of cases, whereas the metastatic lesion was discovered simultaneously at the time of diagnosis in 80% of small cell carcinoma cases.

Lung primary pleomorphic carcinoma is a rare tumor that accounts for only about 0.3% of all lung cancer cases [1]. The incidence is high in elderly men, and the tumor has a causal relationship with cigarette smoking [9]. The tumor is diagnosed after surgical resection in many cases due to the pathological characteristics, but the treatment outcome and prognosis are poor. The response of the tumor to chemotherapy is also low [10].

The patient in the current report was a female cigarette smoker at high-risk of lung cancer, and lung cancer and breast metastasis were observed at the first examination. On imaging, there was no finding that allowed differentiation from primary breast cancer, and the histologic findings on needle biopsy specimens of the lung and mammary gland specimens were also very similar. Therefore, it was impossible to differentiate whether the disease was double cancer (lung and breast cancers), lung metastasis of breast cancer, or breast metastasis of lung cancer until surgical resection of the lung and mammary tumors.

Histologically, the lung tumor had dyscohesive proliferation of spindle, oval, or polygonal cells and scattered multinucleated giant cells. In addition, the tumor had a few foci of squamous cell carcinoma. However, a histologic appearance of squamous cell carcinoma is not identified in the breast tumor. And there were no typical features of conventional breast cancer and ductal carcinoma in situ. We diagnosed this case the breast metastasis of lung cancer, not primary breast cancer.

In the review discussed above [8], it was stated that when breast metastasis was simultaneously present with lung cancer, the diagnosis could not be made before treatment, and the breast was surgically treated at a high rate, as in the present case. Chemotherapy is an option for so-called triple negative breast cancer, but it cannot be performed without a reliable diagnostic basis. Sensitivity to chemotherapy was low in our patient, and the disease rapidly progressed; thus, malignancy was high and the prognosis was poor, as in previous reports.

The white blood cell count in our patient was abnormally high during the disease course, and ultimately elevated again following a transient decrease after chemotherapy. The serum G-CSF level was also high, but G-CSF staining was negative on immunohistological examination. According to Asano et al. [11], the diagnostic criteria for a G-CSF-producing tumor are (1) an increase in peripheral blood white blood cells, mainly mature neutrophils, (2) elevation of serum G-CSF, (3) an increase in neutrophils in tumor-transplanted nude mice, (4) reduction or disappearance of (1), (2), and (3) by tumor excision or treatment, and (5) CSF activity in the culture supernatant of tumor extract. Identification of such tumors is simple based on the serum G-CSF activity and immunostaining of tumor tissue with anti-G-CSF antibody, but our case did not meet the definition of a G-CSF-producing tumor.

In conclusion, this is the first report of breast metastasis of pulmonary pleomorphic carcinoma. The disease aggravated rapidly and differentiation of breast metastasis from primary breast cancer was difficult.
